# Limited Additional Value of a Chest CT in Whole-Body Staging with PET-MRI: A Retrospective Cohort Study

**DOI:** 10.3390/cancers16122265

**Published:** 2024-06-19

**Authors:** Tineke van de Weijer, Wilhelmina L. van der Meer, Rik P. M. Moonen, Thiemo J. A. van Nijnatten, Hester A. Gietema, Cristina Mitea, Jochem A. J. van der Pol, Joachim E. Wildberger, Felix M. Mottaghy

**Affiliations:** 1 Department of Radiology and Nuclear Medicine, Maastricht University Medical Center, P. Debeylaan 25, P.O. Box 5800, 6202 AZ Maastricht, The Netherlands; t.vandeweijer@maastrichtuniversity.nl (T.v.d.W.); liekevandermeer@gmail.com (W.L.v.d.M.); rik.moonen@mumc.nl (R.P.M.M.); thiemo.nijnatten@mumc.nl (T.J.A.v.N.); hester.gietema@mumc.nl (H.A.G.); jochem.vander.pol@mumc.nl (J.A.J.v.d.P.); j.wildberger@mumc.nl (J.E.W.); 2 School of Nutrition and Translational Research in Metabolism (NUTRIM), 6200 MD Maastricht, The Netherlands; 3 School for Oncology and Reproduction (GROW), 6200 MD Maastricht, The Netherlands; 4 School for Cardiovascular Diseases (CARIM), 6202 AZ Maastricht, The Netherlands; 5 Department of Nuclear Medicine, University Hospital, RWTH Aachen University, 52074 Aachen, Germany

**Keywords:** PET/MRI, CT, Oncology

## Abstract

**Simple Summary:**

PET/MRI systems are being installed world-wide for the staging of cancer. As this combines MRI and PET in one system, the application of these systems offers a one-stop-shop for staging in Oncology. However, one of the pitfalls of these systems is that the MRI of the PET/MRI systems is not very adequate for detecting lung nodules. In this study, we show that the most relevant long-nodules suspected of metastasis can be detected with PET; it remains questionable if missing the indeterminate lung nodules are of clinical relevance.

**Abstract:**

Hybrid PET-MRI systems are being used more frequently. One of the drawbacks of PET-MRI imaging is its inferiority in detecting lung nodules, so it is often combined with a computed tomography (CT) of the chest. However, chest CT often detects additional, indeterminate lung nodules. The objective of this study was to assess the sensitivity of detecting metastatic versus indeterminate nodules with PET-MRI compared to chest CT. A total of 328 patients were included. All patients had a PET/MRI whole-body scan for (re)staging of cancer combined with an unenhanced chest CT performed at our center between 2014 and 2020. Patients had at least a two-year follow-up. Six percent of the patients had lung metastases at initial staging. The sensitivity and specificity of PET-MRI for detecting lung metastases were 85% and 100%, respectively. The incidence of indeterminate lung nodules on chest CT was 30%. The sensitivity of PET-MRI to detect indeterminate lung nodules was poor (23.0%). The average size of the indeterminate lung nodules detected on PET-MRI was 7 ± 4 mm, and the missed indeterminate nodules on PET-MRI were 4 ± 1 mm (*p* < 0.001). The detection of metastatic lung nodules is fairly good with PET-MRI, whereas the sensitivity of PET-MRI for detecting indeterminate lung nodules is size-dependent. This may be an advantage, limiting unnecessary follow-up of small, indeterminate lung nodules while adequately detecting metastases.

## 1. Introduction

Positron Emission Tomography (PET) is part of the workup for many cancer diagnoses and has a significant effect on oncological management [1,2]. Most PET scans are acquired on conventional hybrid PET-CT scanners. However, since its introduction in 2010, PET-MRI has been evaluated for clinical applications. Since then, PET/MRI has proved its value in the work-up of cancer diagnosis and staging [3,4]. PET-MRI scans have the advantages of higher soft tissue contrast and radiation dose reduction. Also, for specific tumor types, PET-MRI is superior to PET-CT and more cost-effective [5]. The results vary based on tumor histology [5,6,7]. 

One of the limitations of PET-MRI imaging, however, is its reduced sensitivity for pulmonary nodules when compared to computed tomography (CT) and PET-CT. Therefore, a whole-body PET-MRI is often combined with an additional unenhanced chest CT for lung nodule detection in patients with suspected metastasized disease. This approach may result in the detection of many indeterminate nodules [8]. These nodules often turn out to be benign after follow-up or biopsy [8]. Therefore, the incidental detection of these nodules gives an unnecessary rise to additional follow-up and emotional burden for the patients. Hence, one can question the clinical value of the additional chest CT for staging with PET/MRI, lowering the patient radiation dose.

Some previous studies have already shown that PET/MRI imaging has a high sensitivity for the detection of Fluorodeoxyglucose (FDG)-avid nodules. In the study of Chandarana et al. [9], the sensitivity of PET/MR imaging was 70.3% for all nodules, 95.6% for FDG-avid nodules, and 88.6% for nodules 0.5 cm in diameter or larger. The study of Stolzmann et al. [10] showed a similar detection rate for both (PET-)CT and (PET-)MR imaging (*p* > 0.05). CT revealed 66 pulmonary nodules in 34 of the 40 patients (85%), whereas the MR images showed 58 pulmonary nodules in 33 of the 40 patients (83%). However, these were small studies, with only 34 patients in the study of Chandarana and 40 patients in the study of Stolzmann, respectively [11,12]. Also, it remains questionable whether missing these nodules is a clinically relevant issue since the vast majority may resolve or stay stable without therapy, indicating a benign nature [13]. There have been developments optimizing the MRI protocols for detection of these nodules, further improving the detection of these pulmonary nodules, including breath hold zero-echo time (ZTE) [14,15] and StarVIBE imaging [16,17]. This may contribute to the better detection of lung nodules on PET-MRI.

Therefore, the aim of this retrospective study was to assess the sensitivity of PET-MRI in the detection of lung metastases and indeterminate nodules in a consecutive set of oncological patients scanned in the setting of staging or restaging their disease. In the following sections, we will describe our methodology, present the detailed results, and finally discuss them in the context of the available literature.

## 2. Materials and Methods

This study retrospectively assessed all whole-body PET-MRI exams performed for either staging or restaging of primary cancer at our center between May 2014 and December 2020. The quality of the study was checked with the STROBE -statement checklist (see Supplemental Materials, S1). The study was approved by the Institutional Review Board (IRB, METC number 2022-3503). 

### 2.1. PET-MRI Protocol

Both [^18^F] FDG as well as [^68^Ga]Prostate Specific Membrane Antigen (PSMA) PET scans were included in the study. For the FDG scans, all patients fasted for at least 4 h prior to the examination. When applicable, insulin was discontinued 6 h prior to the examination, with the target blood glucose level verified to be less than 11 mmol/L (200 mg/dL) in all patients. After administration of an intravenous injection of 2–3 MBq/kg of FDG, patients were asked to lay down quietly in a dimly lit room for 45 min and were asked to void prior to imaging.

For the PSMA scans, patients did not have to remain fasted. 3 MBq/kg of PSMA was injected 45 min prior to the scan. Patients were asked to void prior to imaging. For both the FDG and PSMA-PET scans, images were obtained from the skull vertex to the proximal femora.

PET-MRI was initiated on average 51.6 min after tracer injection. A whole-body PET-MRI was performed on a 3.0-Tesla Biograph mMR PET-MRI scanner (Siemens, Erlangen, Germany) with a 4.4-mm PET resolution (full width at half maximum). The PET images were acquired in a 5-min per bed position for both FDG and PSMA. The PET reconstruction was performed with a PSF (point-spread function) algorithm (Siemens HD; 3 iterations, 21 subsets, 4 mm Gauss, matrix size 300) and corrected for attenuation, scatter, randoms, dead-time, and radioactive decay. Two-point DIXON-based attenuation correction maps included soft tissue, fat, lung, and air. Starting in September 2017, bone was added to the attenuation maps using an atlas-based method. PET-MRI images were fused and analyzed using dedicated DICOM software (Syngo.via Client, version V10.3, Siemens Healthcare, Forchheim, Germany).

Quantification of tracer uptake was performed by assessing the standardized uptake value (SUV; measured activity concentration [Bq/mL] × body weight [18]/injected activity [Bq]).

A total body (from skull-base to groin) 2D T2-weighted fast spin-echo image in two planes (sagittal and axial) was performed (TR 2150/ TE 138 ms, echo train length 33, 1 average, (0.98 × 0.98) × 6.50 mm^3^). Furthermore, a T2 weighted coronal series was performed with fat suppression using inversion-recovery (STIR, TR 5990 ms, TE 33 ms, TI 220 ms, 1 average (0.98 × 0.98) × 5.00 mm^3^). These scans were used for the detection of lung nodules in combination with the PET. We did not use the new sequences such as breath hold zero echo time (ZTE) or StarVIBE imaging, as these were not available for all scans. 

Additionally, diagnostic MRI images were performed of the area of interest of the primary tumor (abdominal or pelvic region or of the limbs). However, this data was not included in this study. 

### 2.2. CT Protocol

With all whole body scans, according to standard local protocol, a chest CT was acquired. The chest CT was acquired within 30–60 min before or after the performance of the PET-MRI scan. The scans were performed on a 3rd-generation dual-source CT (DSCT) scanner (Somatom Force, Siemens Healthineers, Forchheim, Germany). The scan range was set from the neck to the upper abdomen. Scan parameters were as follows: quality reference tube voltage was 100 kV, quality reference tube current was 78 mAs, and a pitch of 2. No intravenous contrast injection was applied. Scans were reconstructed at 2 mm axial slices at an increment of 1.5 mm using a sharp (lung) kernel (B57) and at 5 mm using a soft (mediastinum) kernel (B31). 

### 2.3. Image Analysis

All scans were evaluated by 2 readers: a board-certified radiologist and nuclear medicine physician (TvdW, >10 years experience) and a final-year radiology resident with special interest and expertise in cardiothoracic imaging (LvdM, >5 years experience). The readers were blinded to the modalities, patient information, and prior reports and separately analyzed both the PET-MRI images and the CT images in random order. The scans were scored by the patient based on the presence or absence of lung nodules. All nodules were marked on the scans for comparison. Discrepancies between the readers were discussed in a consensus meeting. Lesions were marked based on the image number for correlation between reviewers and the different image modalities. The images of the PET-MRI were assessed for both PET and MRI with Syngo.via (Siemens, Munich, Germany). The CT scans were assessed in Sectra (RIS/PACS version 12, Sectra Imtec AB, Linköping, Sweden). 

The presence of any lung nodule, their greatest dimension, the smallest lung nodule, and the presence or absence of any identifiable tracer uptake were registered. For each nodule marked on CT, the visibility of the nodule was assessed on the PET-MR images. 

### 2.4. Quantitative and Qualitative Analysis

Each reader measured the largest diameter of the smallest detected nodule, independent of the reconstruction plane, on both the PET-MRI examination and the CT study. The outcome of the missed lung nodules was then assessed by re-examining the nodules of the CT on the PET-MRI and comparing their size. All lung nodules were classified as malignant or indeterminate based on follow-up with a chest CT or histological diagnosis. If these lesions were present on prior imaging and unchanged or smaller in follow-up for at least 2 years or resolved without systemic therapy, the lesions were classified as indeterminate. In cases where the nodules resolved upon systemic therapy or showed measurable growth, the lesions were classified as metastases. Also, all nodules with enhanced tracer uptake at primary staging with the typical cannon-ball aspect randomly distributed over the lung parenchyma on CT imaging, were classified as metastases at primary staging, except for one patient with nodules with a typical perilymphatic distribution and known sarcoidosis.

### 2.5. Statistics

#### 2.5.1. Interobserver Variability and Agreement

Interobserver variability in assessing nodule size on CT findings was evaluated using the Weighted Kappa coefficient for ordinal data. The Weighted Kappa coefficient accounts for the degree of disagreement among observers, providing a more nuanced measure of agreement than the simple Kappa coefficient, particularly for ordinal data. A kappa value of less than 0.4 is considered poor agreement. Kappa values of 0.4 to 0.75 are considered moderate to good, and a kappa of >0.75 represents excellent agreement.

#### 2.5.2. Correlation Analysis and Proportional Bias

To assess the correlation between observers’ measurements of nodule size, Pearson’s correlation coefficient was calculated. Additionally, Bland-Altman plots were used to visualize the agreement between two observers, plotting the difference between their measurements against the mean of those measurements. Proportional bias was evaluated by regressing the differences between observers’ measurements on the average of those measurements, assessing whether the discrepancies between measurements were consistent across the range of nodule sizes.

#### 2.5.3. Between-Group Comparisons

For between-group comparisons of continuous data, a two-sample t-test was employed. This test was used to determine if there were significant differences in nodule sizes between groups. Statistical significance was established at a *p*-value of less than 0.05.

#### 2.5.4. Sensitivity and Specificity

The nodules detected on CT and PET/MRI were classified as malignant or indeterminate, as explained above. Within these groups, sensitivity and specificity for the detection of metastases and indeterminate lung nodules were calculated for PET/MRI, and CT. Sensitivity was defined as the proportion of true positives correctly identified by PET-MRI compared with CT. While specificity was defined as the proportion of true negatives correctly identified by PET/MRI compared with CT. 

#### 2.5.5. Statistical Software

All statistical analyses were performed using SPSS (version 23.0.0.0). This software was used for calculating the Weighted Kappa coefficient, conducting correlation and regression analyses for Bland-Altman plots, performing two-sample t-tests, and computing sensitivity and specificity values.

## 3. Results

### 3.1. General Characteristics

In total, 328 patients (216 females and 112 males, age range 25–92 years, mean age 60.8 ± 15.5 years) were included with a known primary malignancy that underwent a clinical whole-body PET-MRI with an additional unenhanced chest CT. The largest group of patients underwent a [^18^F]FDG PET-MRI for staging or restaging of cervical cancer (*n* = 145). The second largest group were patients undergoing staging or restaging of prostate cancer with [^68^Ga]PSMA PET-MRI (*n* = 91). The group of prostate cancer patients was relatively small, as these scans were only acquired in May 2019. Other tumor types included in the cohort are depicted in Table 1.

### 3.2. Inter-Observer Variability

There was a moderate agreement between the observers for the detection of lung metastases for PET-MRI with a kappa (κ) of 0.70 (0.4–0.75 = moderate) and excellent agreement between observers for chest CT with a kappa (κ) of 0.79 (>0.75 = excellent). This indicates that some more inter-observability was introduced using PET-MRI instead of CT. This was even worse for the indeterminate nodules, where there was a moderate agreement for the detection of indeterminate lung nodules on PET-MRI with a kappa (κ) of 0.49 and an excellent agreement on CT with a kappa (κ) of 1.0. 

**Figure 1:**
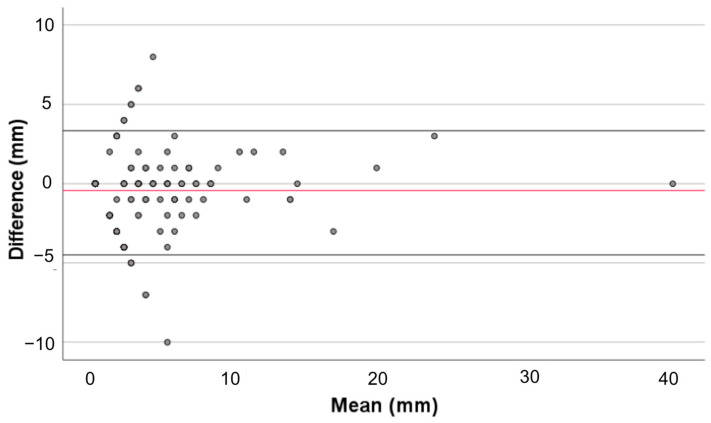
Blant-Altman plot mean differences in nodule size between observers; Overview of mean and differences in nodule size as measured by both observers, with the mean difference-line in black (−0.1458 ± 1.133) and the 95% confidence intervals in dotted lines (2.0742 and −2.3658). There was no proportional bias in the regression analysis of the differences, with a B-value of 0.35 (*p* = 0.532). Missed nodules were not included in this analysis.

Some variation in the nodule size was seen between the observers, though with a high correlation coefficient of 0.96 (95% CI 0.95–0.96) and without statistically significant differences between the observations. This means that there was a random difference in measured size between the observers; however, this variation was not dependent on nodule size, as there was no proportional bias (B-value of 0.35, *p* = 0.532; see Bland Altman plot in Figure 1).

### 3.3. Indeterminate Lung Nodule Detection

**Figure 2:**
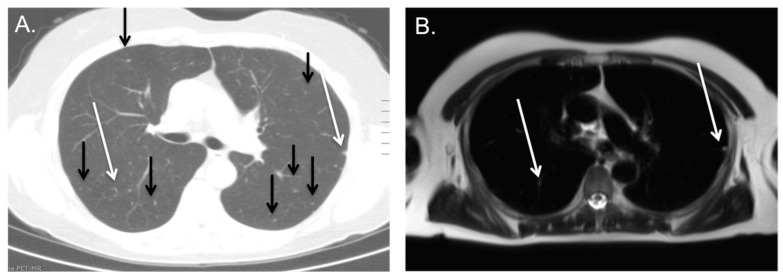
Poor detection of indeterminate Nodules on PET/MRI; patient with a pancreatic carcinoma with many millimetric lung nodules on CT (**A**), of which only a few are visible on MRI (**B**, see white arrows on CT and MRI), but most could not be detected due to their small size (see black arrows on CT). Lung nodules remained stable in follow-up and were classified as benign.

**Figure 3:**
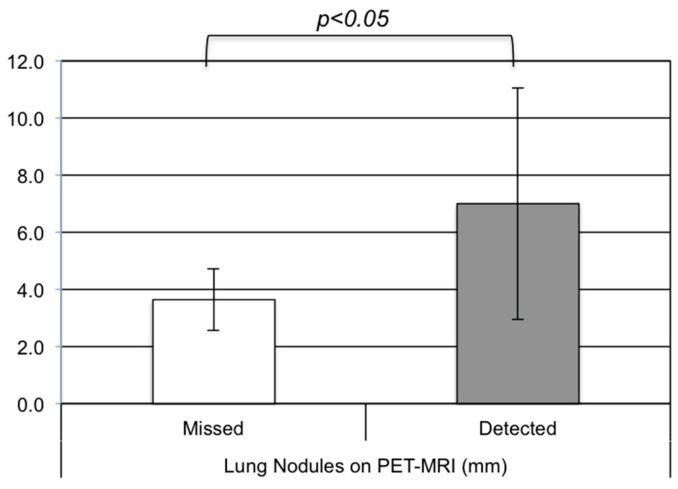
Average smallest size of benign lung nodules.

Within the total population, 100 patients (30%) presented with lung nodules, which were eventually classified as indeterminate. These nodules were defined as indeterminate, when unchanged or disappeared during follow-up without systemic therapy over a period longer than a year (Figure 2). In only 23 cases, the indeterminate nodules were visible on the PET-MRI, yielding a sensitivity of only 23.0% (CI 15.2–32.5%; also see Table 2), meaning that many indeterminate lung nodules were missed in the PET-MRI. Nodules were best visualized on the coronal T2 sequence with fat suppression. Only in two cases, the indeterminate lung noduli show slight tracer uptake on the PET; this was in patients with known sarcoid disease. The average size of indeterminate lung nodules detected on PET-MRI was 7.0 ± 4.1 mm, with a range of 2–15 mm. The smallest size of the missed indeterminate lung nodules on PET-MRI was smaller compared to the mean nodule size of all detected lung nodules on chest CT, with a mean maximum diameter of 3.6 ± 1.1 mm and a range of 2–6 mm (*p* < 0.001, Figure 3). This clearly indicates that the detection of lung nodules was size-dependent. The detection of nodules in the lower lung fields was poor compared to the upper lung fields.

### 3.4. Lung Metastases

**Figure 4:**
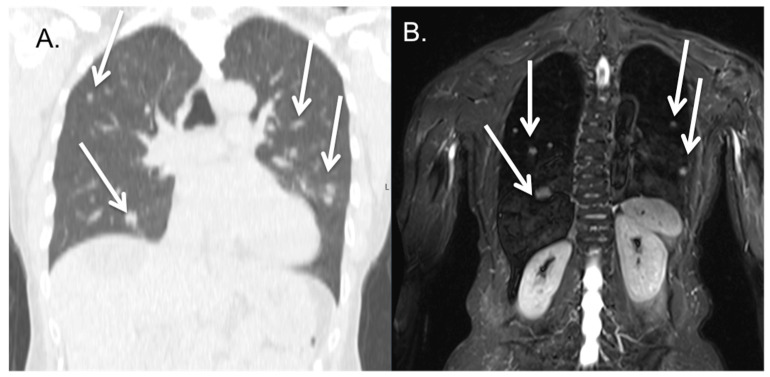
Patient with diffuse lung metastases; diffuse lung metastasis both visible on CT (**A**) and PET-MRI (**B**) in a patient with cervical cancer. Some of the matching lung metastasis are marked by a white arrow on both CT and MRI show matched results for both scans.

**Figure 5:**
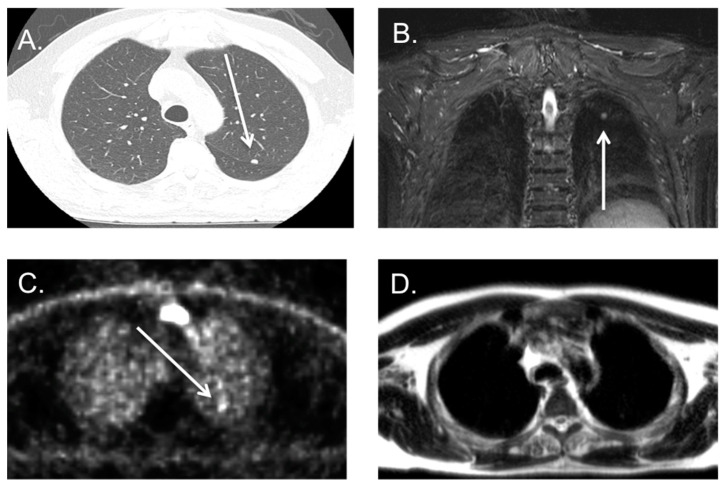
Patient with lung metastasis on both a CT and PSMA PET-MRI scan; [^68^Ga]-PSMA PET-MRI and low dose CT in an 84-year-old male showing a nodule of 6 mm in the left upper lobe on CT, see white arrow (**A**). The nodule is visible on the T2 weighted fat suppressed (STIR) images, see white arrow (**B**) and intense PSMA-avid, see white arrow, (**C**), but hardly visible on the axial T2 sequence (**D**).

**Figure 6:**
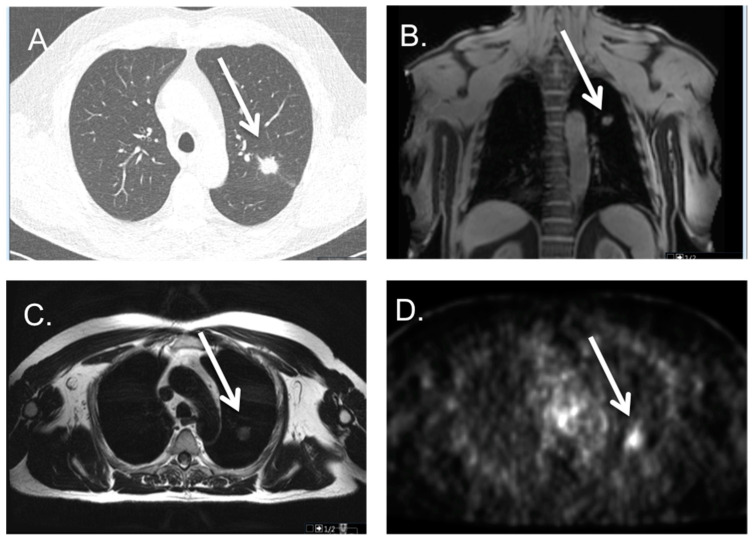
Patient with a primary lung tumor on a PSMA PET-MRI scan; CT and PET-MRI of a patient with spiculated mass in the left upper lobe, visible on CT, see white arrow (**A**) as well as MRI (STIR coronal (**B**) and T2 axial (**C**), see white arrow) and PSMA-PET scan, see white arrow (**D**). This mass was proven by biopsy to be a primary adenocarcinoma of the lung.

Only 6.1% of the patients (20/328) presented with lung metastases. Here, three cases were missed on PET-MRI (Table 3), including 2 patients with vaginal carcinoma and 1 patient with prostate carcinoma (also see Figure 4 and Figure 5). On one of the [^68^Ga]PSMA PET scans, a primary lung carcinoma was found, which was also detected on the additional chest CT (Figure 6). The average smallest size of the detected lung metastases was 11.8 ± 9.3 mm, with a range of 3–40 mm. The average smallest size of the non-detected lung metastases was 6 ± 0.6 mm, with a range of 6–7 mm. Again, the detection of the lung metastases seemed size-dependent; however, due to the small number of patients with lung metastases, this did not reach statistical significance (*p* = 0.34). In the case of diffuse metastatic cancers, the size of the largest metastases was well above the threshold for visibility on MRI as determined by the detection of the indeterminate lung nodules (Figure 6), making them easy to diagnose. Out of the 17 patients with metastases detected on PET-MRI, 16 cases could be detected on PET images, while only 14 cases were detected on MRI images. Here, the PET did aid in the detection of the metastases; for example, Figure 5. The combined sensitivity and specificity of PET-MRI for lung metastases in our cohort were 85% (CI 62.1–96.8%) and 100% (CI 98.8–100.0%), respectively, indicating only a few missed metastases. 

Within the group of patients diagnosed with cervical cancer, only 4 out of 145 patients presented with lung metastases (2.8%). All metastases were detected with both PET-MRI and chest CT. The average size of the metastases was 11 ± 3 mm. Within the group of prostate cancer patients, out of the 91 patients, 7 patients presented with lung metastases (7.7%). As mentioned above, in one patient, the metastases were missed on the PET-MRI scan. The average smallest size of the lung metastases on CT was 8 ± 5 mm. The maximum diameter of the missed pulmonary metastases of prostate carcinoma was 7 mm, possibly below the threshold of detection.

## 4. Discussion

In our study, the sensitivity of PET-MRI for the detection of lung metastases was 85%, using unenhanced chest CT as a reference standard. In the context staging or restaging of metastasized cancer patients with PET-MRI, the additional value of a chest CT is minimal in most cases and could be omitted. However, it should be noted that the incidence of metastases in this cohort was low due to the relatively low likelihood of pulmonary metastases of the most common kinds of cancer in this cohort, namely cervical cancer and prostate cancer. Therefore, these results may not be applicable to cancers with a high likelihood of pulmonary metastases. In such cases, one should exercise caution before omitting the CT chest in addition to PET-MRI imaging.

Only 6.1% of the patients in this study cohort were diagnosed with pulmonary metastases at (re-)staging, whereas the incidence of indeterminate lung nodules was much higher, at 30% in this population. Specifically, the incidence of lung metastases was 2.7% in the group with cervical cancer and 7.7% in the group with prostate cancer. The reported incidence of lung metastases in cervical cancer at primary staging ranges from 4.2% to 7.7% [19,20,21], and within prostate cancer, the reported incidence ranges from 3.6% to 16.1% [22,23]. Hence, compared to the literature, the incidence of lung metastases in cervical cancer was relatively low in our cohort but comparable for prostate cancer. The other cancer groups were too small to assess the incidence. Possibly, the low incidence of cervical cancer can be explained by the fact that screening for cervical cancer has vastly increased in the last decade and more patients are already diagnosed at an earlier stage of disease, lowering the incidence of lung metastases during primary staging [24]. 

In our analysis, we demonstrated a relatively good detection of lung metastases with PET-MRI, showing a sensitivity of 85% and a specificity of 100%. This sensitivity is somewhat lower than the reported sensitivity of MRI for detecting lung metastases when MRI is used alone [25]. However, it is important to note that in the MRI-only setting, dedicated MRI sequences for assessing the lung parenchyma were utilized, unlike in our study, where only fast T2 and a T2 STIR sequence of the lung were acquired. Additionally, the MRI system’s quality in the PET-MRI setup is slightly limited compared to a standalone MRI system, due to interference from the PET component integrated in the PET-MRI system. Adjusting scan protocols and incorporating dedicated lung sequences could potentially enhance lung nodule detection. Other studies confirm that both CT and MRI exhibit high diagnostic accuracy in diagnosing pulmonary nodules, with CT consistently demonstrating superiority over MRI in all studies [26,27,28]. However, this may be at the cost of higher detection of indeterminate lung nodules; however, this remains to be investigated. 

In contrast to MRI alone, the PET-MRI has the added value of the PET-imaging, aiding in the detection of metastases. Although the quality of the PET-detector is slightly lower in a PET-MRI compared to a PET-CT system, the detection of tracer uptake is possible in lesions larger than 4–5 mm [29,30]. Indeed, in our study, some small nodules, smaller than 6–7 mm, were not visible on MRI but, in some cases, could be detected on PET. There was no significant difference in the size of the detected and missed lung metastases; however, this may be due to the small number of lung metastases rather than the sensitivity of the PET. 

The incidence of indeterminate lung nodules in our population was high, with an incidence of 30%. This is comparable to literature with an incidence of 54% in high-risk patients screened for lung cancer [31]. The sensitivity of the PET-MRI for the detection of indeterminate lung nodules in our study is poor, with a sensitivity of 23% and a specificity of 100%, but strongly size-dependent. Also, the detection of nodules in the basal areas of the lower lobes was poor compared to the upper lung fields. This can be explained by movement artifacts of the diaphragm and the lower PET sensitivity due to smearing artifacts in the lower lung segments [32]. Notably, we only used non-enhanced CT in this study. Studies do collectively suggest that while contrast enhancement may improve the characterization of lung nodules (e.g., distinguishing between benign and malignant lesions), it does not significantly enhance the sensitivity for detecting lung nodules compared to non-enhanced CT. Furthermore, contrast enhancement can sometimes obscure small nodules due to the enhancement of surrounding structures and potential artifacts [33,34,35].

The detection of initial indeterminate lung nodules resulted in additional follow-up scans with CT in approximately 60% of this population, which can be considered a substantial burden. Missing these indeterminate nodules may result in less work-up but also less stress for the patients, which can be an argument to avoid the additional chest CT. Furthermore, the finding of isolated pulmonary metastases in patients with prostate cancer and cervical cancer is rare, while pulmonary metastases are almost always accompanied by metastases in other organs (92.5%) [23], more easily detected at PET-MRI. Therefore, one can consider limiting the application of this additional chest CT in this group to patients with metastases elsewhere.

Other studies comparing the performance of PET-CT and PET-MRI in the detection of pulmonary nodules also show that PET-MRI is inferior to PET-CT [11]. However, missed lung nodules rarely lead to a change in TNM classification [12]. In the recent study of Martin et al. [7], only in 8 out of 1003 cases were malignant lung nodules missed, which only led to a change in TNM classification in 0.5% of these cases. In the study of Biondetti et al. [36], studying primary abdominal malignancies with PET-MRI, the sensitivity of the detection of indeterminate lung nodules was indeed low, since as much as 22.3% of missed nodules demonstrated growth after follow-up, indicative of metastases. However, this did not impact clinical management in this study group, as all cases had advanced extra-thoracic cancer. The study by Raad et al. [13] and Sawicki et al. [12] showed that up to 97% of the lung nodules missed on PET-MRI were benign. Therefore, the impact on therapeutic management of the detection of these nodules is questionable, while missing indeterminate nodules may reduce stress and costs due to fewer follow-up CTs.

## 5. Conclusions

Comparing hybrid PET-MRI and unenhanced chest CT for the detection of lung metastases revealed a high sensitivity for the hybrid approach. In the setting of staging or restaging of metastasized cancer patients using PET-MRI, the additional value of a chest CT is in most cases scarce and could certainly be omitted in those with limited disease and a low likelihood of lung metastases, decreasing on the one hand the (in any case low) radiation dose but, more importantly, lowering patients emotional stress and burden of follow-up of indeterminate lung nodules. 

## Figures and Tables

**Table 1:** Tumor entities and patient characteristics included in the study.

Primary Tumor	Number of Patients	Male (*n*)	Female (*n*)	Age (Years)
Cervix carcinoma	145	0	145	52.6 ± 16.0
Prostate carcinoma	91	91	0	73.0 ± 7.1
Other gynecological tumors *	30	0	30	63.1 ± 12.4
Sarcoma and desmoid tumors	17	3	14	56.0 ± 17.3
Colorectal carcinoma	19	9	10	67.1 ± 10.5
Breast cancer	8	0	8	62.0 ± 9.9
Other **	18	9	9	55.3 ± 15.2
Total	328	112	216	60.8 ± 15.5

* including vulval and vaginal carcinomas and ovarian carcinoma; ** head/neck tumors including adenocarcinoma, squamous cell carcinoma, thyroid carcinoma, Merkel cell carcinoma, melanoma, Pancreas carcinoma, cholangiocarcinoma, Esophageal carcinoma, and Thymoma.

**Table 2:** Detection of indeterminate lung nodules with PET-MRI (in patients without metastases, *n* = 308).

		CT		
		Indeterminate Lung Nodules	No Lung Nodules	
PET-MRI	Indeterminate lung nodules	23	0	23
	No Lung Nodules	77	208	285
		100	208	308
		**Value**	**95% CI**	
**Sensitivity**		23.0%	15.2–32.5%	
**Specificity**		100.0%	98.2–100.0%	
**Positive Likelihood Ratio**		-	-	
**Negative Likelihood Ratio**		0.77	0.69–0.86	
**Disease prevalence**		32.5%	27.27–38.01%	
**Positive Predictive Value**		100.0%	-	
**Negative Predictive Value**		73.0%	70.82–75.04%	
**Accuracy**		75.0%	69.77–79.74%	

**Table 3:** Detection of lung metastases with PET-MRI in the total population.

		CT		
		Metastases	No Metastases	Total
PET-MRI	Metastases	17	0	17
	No Metastases	3	308	311
	Total	20	308	328
		**Value**	**95% CI**	
**Sensitivity**		85.0%	62.1–96.8%	
**Specificity**		100.0%	98.8–100.0%	
**Positive Likelihood Ratio**		-	-	
**Negative Likelihood Ratio**		0.15	0.05–0.43	
**Disease prevalence**		6.1%	3.76–9.26%	
**Positive Predictive Value**		100.0%	-	
**Negative Predictive Value**		99.0%	97.31–99.66%	
**Accuracy**		99.1%	97.35–99.81%	

## Data Availability

The datasets used and/or analyzed during the current study are available from the corresponding author on reasonable request.

## Supplementary Materials

The following supporting information can be downloaded at: https://www.mdpi.com/article/10.3390/cancers16122265/s1, S1: STROBE-checklist.
